# Bandgap Engineering of Ga_2_O_3_ by MOCVD Through Alloying with Indium

**DOI:** 10.3390/nano16020093

**Published:** 2026-01-12

**Authors:** Md Minhazul Islam, A. Hernandez, H. Appuhami, A. Banerjee, Blas Pedro Uberuaga, F. A. Selim

**Affiliations:** 1 Department of Physics and Engineering, Bowling Green State University, Bowling Green, OH 43403, USA; 2 Materials Science and Engineering, School for Engineering of Matter, Transport and Energy, Arizona State University, Tempe, AZ 85287, USA; 3 Department of Metallurgical and Materials Engineering, Indian Institute of Technology Jodhpur, Karwar 342030, Rajasthan, India; 4 Materials Science and Technology Division, Los Alamos National Laboratory, Los Alamos, NM 87545, USA; blas@lanl.gov

**Keywords:** semiconductor, wide bandgap material, density functional theory, tuning electron mobility, electronic transport, hydrogen doping, density of states

## Abstract

Ga_2_O_3_ and In_2_O_3_ are vital semiconductors with current and future electronic device applications. Here, we study the alloying of In_2_O_3_ and Ga_2_O_3_ (IGO) and the associated changes in structure, morphology, band gap, and electrical transport properties. Undoped films of IGO were deposited on sapphire substrates with varying indium (In) percentage from zero to 100% by metal-organic chemical vapor deposition (MOCVD). Some films were annealed in H_2_ to induce electrical conductivity. The measurements showed the optical band gap decreased by adding In; this was confirmed by density functional (DFT) calculations, which revealed that the nature of the valence band maximum and conduction band minimum strongly relate to the chemistry and that the band gap drops by adding In. The as-grown films were highly resistive except for pure In_2_O_3_, which possesses p-type conductivity, likely arising from In vacancy-related acceptor states. N-type conductivity was induced in all films after H-anneal. DFT calculations revealed that the presence of In decreases the electron effective mass, which is consistent with the electrical transport measurements that showed higher electron mobility for higher In percentage. The work revealed the successful band gap engineering of IGO and the modification of its band structure while maintaining high-quality films by MOCVD.

## 1. Introduction

Ternary and quaternary oxide semiconductors have become the focus of intense investigations by materials researchers [1,2,3,4,5,6,7,8] as thin film transistors (TFTs) based on ternary oxide semiconductors have shown superior characteristics, especially in the display industry, owing to their superior characteristics over polycrystalline or amorphous silicon [9,10]. Due to the wide bandgap, TFTs based on oxide semiconductors exhibit low leakage current combined with modest mobility. Some of the oxide semiconductors that have been studied for TFT applications are In-Ga-Zn-O (IGZO) [11,12], Zn-Sn-O (ZTO) [13], Zr-In-Zn-O [14], and Zn-Ba-Sn-O (ZBTO) [15]. In particular, amorphous IGZO (a-IGZO) is an excellent material with a field effect mobility of <20 cm^2^/Vs [16]. Oxide semiconductors such as a-IGZO and a-IZO have also been studied for phototransistor applications and as sensor materials [17,18]. However, to build solar blind photodetectors, larger bandgap materials are needed. In_2_O_3_ is a transparent conducting oxide (TCO) with a wide optical bandgap of ~3.7 eV [19]. It has high mobility due to the large number of oxygen vacancies in the crystal; however, this can be detrimental for TFTs due to a negative shift in threshold voltage [20,21]. Several studies of doping In_2_O_3_ by gallium, hafnium, or silicon, introduced to reduce the number of oxygen vacancies, were reported [10,22,23]. Since the In-O bond dissociation energy (346 kJ/mol) is lower than the others mentioned above (for example, Ga-O with a bond dissociation energy of 374 kJ/mol), replacing indium with Ga can reduce the number of oxygen vacancies and improve the TFT’s performance [22]. Moreover, the size of Ga in the lattice is very similar to In, which allows for the incorporation of Ga in the In_2_O_3_ structure or In in the β-Ga_2_O_3_ structure without significant distortion or carrier perturbation.

β-Ga_2_O_3_ is also a transparent oxide with an ultrawide bandgap of ~4.9 eV. The material has recently received a great deal of attention as a potential material for high-power devices (e.g., Field Effect Transistors (FETs)), solar blind photodetectors, Schottky barrier diodes, and TCOs [24,25,26,27,28,29,30], and because of its interesting electronic transport properties [31]. To achieve the desired electronic configuration at interfaces, the tunability of the bandgap and crystal structure is needed. Moreover, the mixing of different cations in the crystal structure can facilitate the possibility of incorporating shallow donors and acceptors with good mobility and carrier concentration by altering the band structure. There have not been many successes toward realizing p-type β-Ga_2_O_3_, which is crucial for bipolar device applications [32,33]. However, lately, our group has reported the very first p-type conductivity in β-Ga_2_O_3_ with high free carrier concentration at room temperature by altering intrinsic defects with hydrogen [27]. However, the hole mobility was critically low due to the characteristic features of the valence band in β-Ga_2_O_3_. Recent reports showed that p-type conductivity can be increased by reducing compensating donor defects (e.g., oxygen vacancies) [34,35]. Yet, the carrier concentration and mobility were low, and the results were non-reproducible.

Conventional density functional theory (DFT) combined with hybrid functional calculations revealed that the valence band minimum (VBM) of Ga_2_O_3_ primarily consists of oxygen (O) 2p orbitals [36], which are characterized by weak dispersion, high density of states, and large hole effective masses, a behavior commonly observed in wide-bandgap oxide materials [36]. Such electronic features promote the formation of localized oxygen holes known as polarons [37,38], which contribute to the hopping mechanism of hole conduction. Consistent with this, Varley et al. [36]. reported the presence of self-trapped holes in Ga_2_O_3_, in agreement with experimental findings. Materials with deep VBMs dominated by O 2p states are unfavorable for achieving p-type conductivity due to poor hole mobility [39]. Moreover, Kyrtsos et al. [39] demonstrated that all the potential acceptor dopants (X = Li, Na, K, Be, Mg, Ca, Cu, Au, and Zn) result in deep acceptor levels and are unable to contribute to the p-type conductivity in Ga_2_O_3_. One possible pathway to achieve more delocalized valence band and shallow acceptor levels is alloying the β-Ga_2_O_3_ with an element that will not significantly lower the bandgap but will, however, increase the valence band energy just enough to ionize the dopants at room temperature. Further, the replacement of the Ga site will facilitate the formation of a valence band where the top has enhanced contribution from metal orbitals, leading to the increased mobility of holes. Recently, theoretical calculations revealed that bismuth (Bi) can modify the valence band of TSO through the contribution of Bi 6s orbitals that are significantly more delocalized and create an intermediate band above the valence band [33,40]. However, the large mismatch of Bi^3+^ (117 pm) and Ga^3+^ (76 pm) ions is expected to create large crystal distortion and carrier perturbation. In addition, the most stable form of Bi_2_O_3_ at low temperature is α-Bi_2_O_3_, which has a direct bandgap lower than 3 eV [41]. As a result, alloying β-Ga_2_O_3_ with Bi will significantly reduce the bandgap of the resulting alloy, which is not desired for high-power device applications. Moreover, the lack of a suitable precursor for Bi, especially a metal-organic precursor for metal-organic chemical vapor deposition (MOCVD), makes it impractical for the epitaxial growth of thin films [42]. In (94 pm) has a comparable ionic radius with Ga^3+^, and In_2_O_3_ has a dipole permitted optical bandgap of ~3.7 eV, which makes it more suitable for Ga_2_O_3_ alloying. In addition, high-quality metal-organic precursors are readily available for In. From the aforementioned discussion, it is clear that alloying Ga_2_O_3_ with In or In_2_O_3_ with Ga may improve their electronic properties and solve some of their associated challenges. Further, the availability of high-quality metal-organic precursors for In and Ga allows their deposition by MOCVD; moreover, the advancement of β-Ga_2_O_3_ deposition related MOCVD technique is supported by several studies, for example, Alema et al. demonstrated that close-coupled showerhead (CCS) MOCVD can produce high-purity β-Ga_2_O_3_ films with controlled crystallinity and excellent scalability to large areas [43]. Further. their subsequent works demonstrated the capability of tuning the oxidant chemistry with MOCVD, which significantly reduces unintentional carrier density and improves film quality [44], highlighting the broad processing window accessible for β-Ga_2_O_3_–related thin films with MOCVD. In this work, we investigate alloying of β-Ga_2_O_3_ with In with different percentages from 0 to 100% by depositing high-quality thin films by MOCVD and study how alloying modifies their band gap energy, crystal structure, morphology, and electrical transport properties. The study demonstrates the successful engineering of the Ga_2_O_3_ band gap from 4.9 to 3.6 eV. All the undoped films were highly resistive; however, pure In_2_O_3_ exhibits p-type conductivity.

## 2. Materials and Methods

Films were deposited in a closed-coupled showerhead MOCVD reactor, where the precursors and gases were uniformly introduced in a vertical geometry [29]. The gas supply lines of the MOCVD are made of stainless steel and are maintained at 550 °C during growth to prevent the deposition of metal precursors on the lines. Various precision mass flow controllers and manual/automatic valves are used to precisely control the pressure and mass flow rates of the gases at different points of the supply system. N_2_ gas is used as a purging gas. Argon is used as the carrier gas to carry the metal-organic precursors from the stainless-steel bubblers to the reactor. Tri-methyl Indium, tri-ethyl gallium, and pure oxygen were used as the precursor and reactant for indium, gallium, and oxygen, respectively. The bubblers are kept in automated bath temperature controllers to precisely control the bubbler temperature, which ultimately affects the mass flow rates of the precursors in the carrier gas. The showerhead is positioned above the heated wafer to enable a rapid, uniform diffusion of gas molecules onto the rotating, heated wafer. The wafer is inductively heated with an RF heater, capable of sustaining a temperature above 1000 °C during growth. Thermocouples are used to monitor the temperature of the substrates. The rotating shaft and heating coil are connected to a motor and RF power source via vacuum feedthroughs. Two water heat exchangers are used to control the reactor wall and pump casing temperature. The system runs using Imperium Control Software Modules by Agnitron Technology. A multistage vacuum pump equipped with a turbomolecular pump designed by EBARA is used to control the reactor pressure. Nitrogen gas is used to provide a ballast to the vacuum pump. This EBARA pump assembly can generate many levels of vacuum, from intermediate (~10^−2^ Pa) up to ultra-high levels (~10^−8^ Pa), giving the gas molecule a preferential diffusion vacuum. The unreacted gas mixtures from the reactor go through a charcoal filter followed by a scrubber before being released to the exhaust system. In this study, double-sided polished (epi-polished) C-plane sapphire single crystals with a 2-inch diameter and 500 μm thickness were used as substrates. The substrates were cleaned before the growth using a spin coater while spraying ethanol, isopropanol, and deionized water at different intervals, and dried with Nitrogen gas to produce high-quality epitaxial films [45]. All the InGaO (IGO) films in this study were grown at 760 °C for a deposition duration of 3 h. The IGO films had a thickness of approximately 185 nm, whereas the Ga_2_O_3_ film was thinner, at ~100 nm, due to the absence of the indium precursor in the reaction chamber.

X-ray diffraction (XRD) spectroscopy was performed using a Rigaku diffractometer. The samples were irradiated by the Kα line of Cu, and the Kβ line was minimized using a β filter (Ni). The 2θ scan was performed keeping the sample and source fixed while the detector was scanned from 10° to 90° at a scanning speed of 2°/min. Optical absorption spectroscopy was performed at room temperature using a dual-beam PerkinElmer ultraviolet–visible–near-infrared (UV-Vis-NIR) spectrometer, which covers the wavelength range from 1100 nm to 190 nm. More details about the setup can be found in [46]. The scan speed and slit width for the experiments were set as 240 nm/min and 1 nm, respectively. Hall-effect measurements and the temperature-dependent transport measurements were carried out using a customer MMR Hall-effect system equipped with a Joule–Thomson (JT) refrigerator [47]. A commercial aluminum-coated silicon atomic force microscopy (AFM) probe was used to obtain the topographic and phase images of the samples. The force constant of the cantilever was 50 N/m, and the radius of curvature of the AFM tip was 8 nm. A full-scale frequency sweep was performed to tune the AFM tip, and the resonance frequency of the cantilever was found at 170 kHz. The uniform conical shape of the tip fits the requirement to obtain topographic images of the samples. A high-precision closed-loop AFM scanning module (PicoSPM, Agilent) was used for imaging the 10 × 10 µm2 area of each sample in tapping mode. The pixel density of each scan was fixed at 512 × 512. All AFM images were analyzed, and average surface roughness values were extracted using WS×M 5.0 software.

## 3. Results and Discussion

### 3.1. X-Ray Diffraction (XRD)

**Figure 1:**
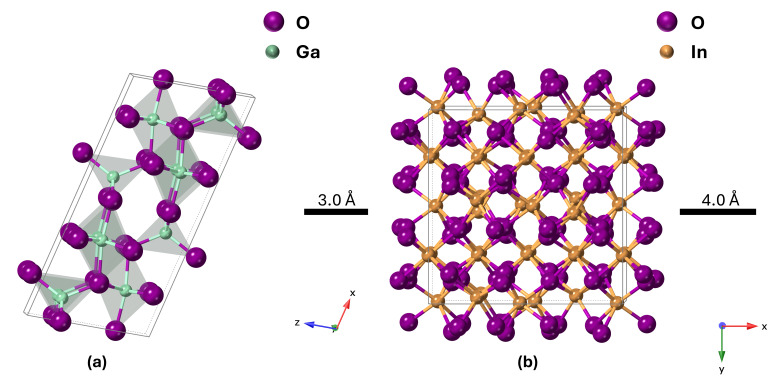
Unit cell of (**a**) monoclinic β-Ga_2_O_3_ and (**b**) bixbyite In_2_O_3_. Purple spheres represent oxygen, light blue represent Ga, and gold represent In.

**Figure 2:**
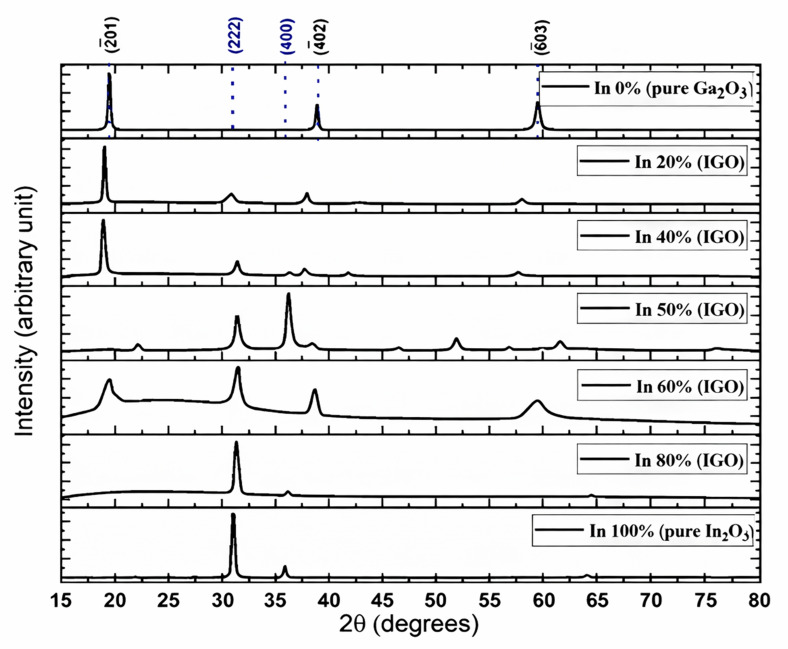
XRD of the films grown on sapphire substrates by MOCVD technique (The reflections from the sapphire planes were removed for simplicity; Black indices represent diffractions associated with Ga_2_O_3_ planes, and blue are from In_2_O_3_.

The most stable polymorph of pure Ga_2_O_3_ (β-Ga_2_O_3_) has a monoclinic crystal structure, while pure In_2_O_3_ prefers the bixbyite crystal structure (Figure 1). However, there is not sufficient experimental research on the IGO structure. Peelaers et al. [48] reported that the monoclinic IGO structure has a lower formation enthalpy up to 50% indium, while the bixbyite structure is lower beyond 50%. However, their study considers only two structures, where a small amount of In in Ga_2_O_3_ preserves a monoclinic structure and a small amount of Ga in In_2_O_3_ preserves a bixbyite structure. Swallow et al. [49] identified three phases of PLD-grown IGO alloy: monoclinic structure at low In content, bixbyite at high In content, and a hexagonal phase in between. Here, we performed 2θ scans on the IGO films grown by the MOCVD technique to investigate the crystal structure. Figure 2 shows the diffraction pattern of the films for different percentages of In from 0 to 100. The plot shows that pure Ga_2_O_3_ is solely (-201) oriented. However, pure In_2_O_3_ exhibits reflections originating from (222) and (400) planes at 2θ = 30.95 and 35.93 degrees, respectively [50].

The plot shows that the films exhibit reflections from Ga_2_O_3_ planes up to 40% in content, confirming the preservation of monoclinic structure. However, the (-201) peak shifts gradually toward a lower angle from 2θ = 19.42 to 18. 89 degrees as the indium atomic percent is increased. In^3+^ has a larger ionic radius (94 pm) than the Ga^3+^ ions (76 pm); therefore, the incorporation of In in the monoclinic structure would increase the unit cell volume. As a result, the reflections move toward smaller angles. This also confirms the incorporation of In in the Ga_2_O_3_ lattice. When the Indium percentage exceeds 40%, the reflections from β-Ga_2_O_3_ weaken and eventually disappear, and after the In content exceeds 50%, the XRD starts to display bixbyite-type In_2_O_3_ peaks, providing a clear indication of the phase transition. The reduction in the monoclinic peaks’ intensities near 40–50% is likely due to the coexistence of both monoclinic and bixbyite domains, accompanied by compositional inhomogeneity and local strain relaxation, contrasting the density functional theory (DFT) study performed by Peelaers et al. [48], who reported that the monoclinic structure has a lower formation enthalpy at 50% In atomic concentration. However, the results are consistent with the findings that are beyond 50% In, as well as with Swallow et al. [49], who reported the energetic favorability of the bixbyite phase over the monoclinic phase beyond ~45% In concentration. The (222) reflection from 2θ = 31.04 degrees in bixbyite In_2_O_3_ moves toward a larger angle as the Ga atomic percent is increased from 0 to 50%. The incorporation of smaller Ga atoms into the In_2_O_3_ lattice leads to lattice contraction, confirmed by the decreased interplanar spacings of the 222 plane with Ga addition calculated by Bragg’s equation, as shown in Table 1. On the other hand, the addition of In into Ga_2_O_3_ results in lattice expansion due to the substitution of larger In atoms for Ga, reducing the lattice spacing (-201). The FWHM of the diffracted peaks for the 60% In concentration film is slightly broader than that of the other films, which is inversely proportional to the grain size.

### 3.2. UV-Visible Absorption Spectroscopy

Optical transmission properties of the films were investigated using UV-Vis absorption spectroscopy. Monoclinic β-Ga_2_O_3_ is an ultra-wide bandgap material with an energy bandgap larger than 4.5 eV. As a result, the material is transparent all the way up to the UV region of electromagnetic radiation. The energy of the absorption edge was reported to be different in the directions in the crystal due to the anisotropy [51,52]. Moreover, experimental reports have shown that the bandgap (Eg) for single crystals (4.52–4.6 eV) is smaller than for films (4.75–5.0 eV) [51,52,53]. Analysis of their band structure published results revealed that the valence band maximum occurs at two different points on the momentum axis, Γ and M, while the conduction band minimum occurs at Γ. Theoretical calculations have revealed that the direct bandgap is 3.40 eV at Γ and the indirect bandgap at M is 3.37 eV, resulting in a difference of 0.03 eV between the direct and indirect bandgaps of β-Ga_2_O_3_ [54]. Experimental results also found a similar difference of 0.05 eV.45. Therefore, β-Ga_2_O_3_ can be considered as a direct bandgap material. On the other hand, bixbyite In_2_O_3_ has a dipole-allowed bandgap of ≈3.7 eV, while indirect-forbidden transitions have also been observed with an energy gap of 2.619 eV [55]. Therefore, In_2_O_3_ has a wide bandgap, even though it is significantly lower than that of β-Ga_2_O_3_, creating the opportunity of tuning the bandgap of the IGO within a wide range.

**Figure 3:**
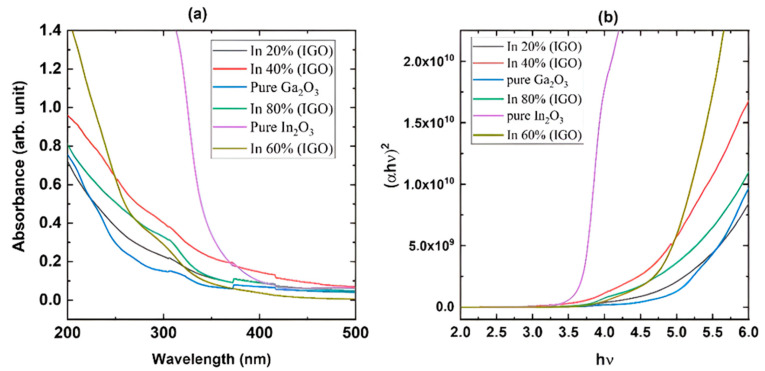
UV-Vis absorption spectroscopy of the films grown on sapphire substrates at 760 °C: (**a**) absorption spectra and (**b**) Tauc plot to calculate optical bandgap.

**Figure 4:**
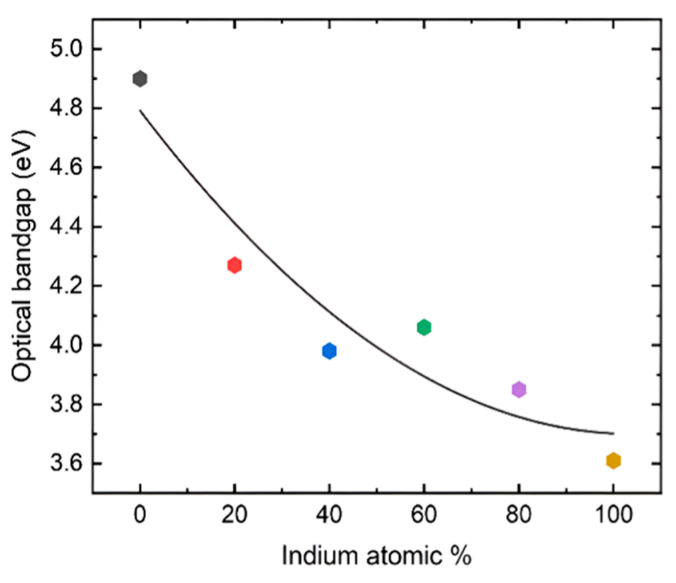
Optical bandgap vs. composition of the films grown on the sapphire substrates at 760 °C using the MOCVD technique. The color of the points is consistent with the UV-Vis absorption spectra and Tauc plots in Figure 3.

Figure 3a shows the absorption measurements of the films grown on sapphire substrates, which reveal that the films are highly transparent in the visible region. The plot also reveals that the pure β-Ga_2_O_3_ has the shortest cut-off wavelength, while pure In_2_O_3_ has the longest. The cut-off wavelength gradually moves toward the longer wavelength as the concentration of In is increased, confirming the growth of the films with the desired composition. Pure β-Ga_2_O_3_ exhibits an absorption shoulder at longer wavelengths due to the anisotropy of the bandgap in different directions, which gradually disappears as it changes to the bixbyite structure. Figure 3b shows the Tauc plot used to calculate the optical bandgap. Figure 4 shows the change in optical bandgaps of the films vs. composition. The band gaps of the films were calculated using the Tauc plot [56,57] which relates the absorption coefficients to the light frequency via the following equation.

(1) $$\alpha h\nu =B{\left(h\nu -E_{g}\right)}^{r}$$

where E_g_ is the optical bandgap of the sample, α is the absorption coefficient, and r is a constant that has a value of ½ for a direct band gap and a value of 2 for an indirect band gap. r was taken as ½ since this is a direct band gap material. Figure 4 shows that the pure β-Ga_2_O_3_ has an ultra-wide bandgap of 4.9 eV, which matches the reported values for films. On the other hand, In_2_O_3_ exhibits a dipole-allowed bandgap of 3.61 eV that also complies with the reported value. All the other values for the films exhibit band gaps with intermediate values that gradually decrease as the In content is increased. However, the film grown with 60% indium content shows a slightly higher bandgap than the 40% composition, possibly due to the polycrystalline nature with smaller grain size as shown in the XRD plot (Figure 2). The FWHM of the diffracted peaks for 60% In concentration is slightly larger than the other films, which is inversely proportional to the grain size.

### 3.3. Atomic Force Microscopy

**Figure 5:**
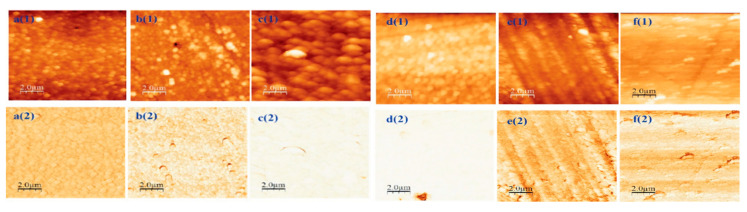
AFM topographic and phase images of the films grown with different compositions on sapphire substrates at 760 °C. **a(1)**–**f(1)** are the topographical and **a(2)**–**f(2)** are the phase images of the films grown with 0–100 atomic % In, respectively.

To understand the effects of alloying on the surface morphology of the films, which can significantly impact the electrical transport properties, AFM measurements were performed, as shown in Figure 5. The figure displays the topographic and phase images of the 10 × 10 μm^2^ scan of the films grown on sapphire substrates at 760 °C. The films exhibit varying surface roughness, with the lowest root-mean-square (rms) roughness of 4.57 nm for the pure Ga_2_O_3_ and the largest rms roughness of 33.06 nm for the 20% In concentration. Pure In_2_O_3_ exhibits a roughness of 6.21 nm, which also implies that the ternary alloys tend to produce larger feature sizes than the binary structures. Most of the films, except those that have grown by 80%, exhibit an island-type surface feature. The film grown with 80% In produces a terrace-like surface morphology. Pure In_2_O_3_ exhibits a flatter morphology than the other films. All the samples exhibit homogeneous films, as evident from the phase images.

### 3.4. Hall Measurements

Electrical parameters of the films were determined by Hall-effect measurements. Table 2 shows the electrical parameters of the films grown on sapphire substrates. Most of the as-grown films exhibit high electrical resistivity, except for pure In_2_O_3_. Pure Ga_2_O_3_ has a carrier density of 3.02 × 10^10^ cm^−3^ with a resistivity of 1.01 × 10^4^ Ω·cm, which is an indication of a good-quality film with low n-type background conductivity. Undoped Ga_2_O_3_ has been reported to be n-type with carrier concentration as high as 2 × 10^18^ cm^−3^ due to unintentional doping or shallow point defects [24,27,58]. This high unintentional carrier concentration makes it problematic to use in devices, especially in the drift region, due to the low breakdown voltage. However, the MOCVD method used in this work allows better control of native defects and impurities.

IGO films with 20 and 40% In exhibit even lower carrier concentrations of 2.92 × 10^9^ and 6.07 × 10^8^ cm^−3^, respectively. IGO films with 60 and 80% In have extremely low electrical conductivity, beyond the sensitivity of the instrument. However, pure In_2_O_3_ shows a very different behavior.

The film shows p-type conductivity with a carrier density of 6.15 × 10^17^ cm, resistivity of 0.65 Ω·cm, and Hall mobility of 15.69 (cm^2^/Vs). As-grown In_2_O_3_ usually exhibits n-type conductivity with high electron concentration, probably due to oxygen vacancies, hydrogen interstitials, or hydrogen-vacancy complexes [59]. However, the oxygen-rich growth environment used here is probably responsible for a high concentration of In vacancies or oxygen interstitials, which imparts high p-type conductivity to the film [60,61].

Hydrogen is known to be a common impurity in oxide semiconductors, including Ga_2_O_3_ and In_2_O_3_. It is known to occupy the interstitial or substitutional sites or create complexes with other intrinsic/extrinsic defects, passivating compensating acceptors [62], or acting as shallow donors or acceptors in oxide semiconductors [27,59,63,64]. To understand the effects of hydrogen in IGO films, we diffused hydrogen into some of the IGO films, and their electrical parameters were studied.

**Figure 6:**
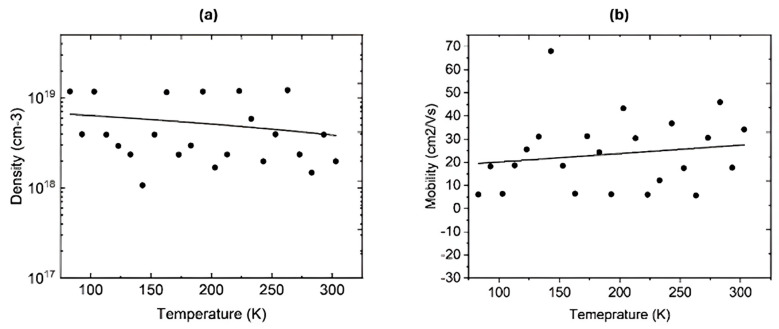
(**a**) Free carrier density of the IGO film (In 80%) at variable temperatures. (**b**) Hall electron mobility in IGO film (In 80%) at variable temperatures. The data points are scattered due to noise in the signals caused by the long wires connecting the Hall.

Hydrogen was diffused at 400 °C in a closed quartz ampoule filled with hydrogen at 580 torr for 3 h. After the diffusion, Hall measurements were performed on two samples at room temperature and at variable temperatures. Table 3 shows the electrical parameters of the two IGO films at room temperature. These samples were deliberately selected because IGO with 20% In lies near the Ga_2_O_3_-rich monoclinic regime, and IGO with 80% In lies in the In_2_O_3_-rich bixbyite regime, respectively. Therefore, they exhibit the largest differences in crystal structure, bandgap, and donor behavior across the IGO alloy series. It is evident that the free carrier density has increased immensely after the hydrogen incorporation. Both samples were highly resistive before hydrogen diffusion, and their resistivity decreased to 0.59 and 0.089 Ω·cm after hydrogen incorporation. Both samples exhibit n-type conductivity. The dramatic decrease in resistivity upon hydrogen diffusion suggests that hydrogen acts as shallow donors, passivating and compensating acceptor-like defects such as gallium vacancies (V_Ga_). This finding is in good agreement with previous studies, which have observed similar hydrogen-induced n-type conductivity in Ga_2_O_3_ and In_2_O_3_ [27,59]. The large resistivity change in low In thin films should be attributed to the passivation of more acceptor-type defects, as Ga-rich IGO lattice tends to host more V_Ga_. In contrast, In-rich films already consist of a higher intrinsic carrier density, thereby minimizing the relative impact on conductivity by hydrogen diffusion. Low-temperature Hall-effect measurements were performed on the IGO film grown with 80% In to study the ionization of the donors. The system can examine temperatures from 80 K to 600 K. Figure 6a shows the free carrier density at variable temperatures, revealing that the carrier freeze-out happens at a temperature lower than 80 K due to the shallow nature of the donor states [55]. This explains why the carrier density remains similar down to 80 K. Figure 6b shows the Hall mobility of electrons at variable temperatures, which also remains somewhat constant up to 80 K.

### 3.5. Density Functional Theory and Band Structure Calculations

To better understand the impact of In on the band structure of Ga_2_O_3_, we performed DFT of both pure Ga_2_O_3_ and mixed IGO. DFT calculations were performed using VASP (Vienna Ab initio Simulation Package) [65,66]. A supercell of 160 atoms (32 formula units) was considered in these calculations. The Special Quasi-random Structures (SQS) [67], an approach to mimic random distributions of atoms in crystalline solids, was used to create the In-doped structure. The Brillouin zone was sampled using 3 × 3 × 3 and 7 × 7 × 7 meshes of Monkhorst–Pack *k*-points for optimization and electronic structure calculations, respectively. The core electron behavior and the interaction between the valence electrons and the ions were described by the projector augmented wave method (PAW) [68,69] The Perdew–Burke–Ernzerhof (PBE) form of the generalized gradient approximation (GGA) was employed as the exchange-correlation functional to obtain the optimized ground state structure [70]. The valence electrons were described by a plane-wave basis set with a converged energy cut-off of 520 eV.

**Figure 7:**
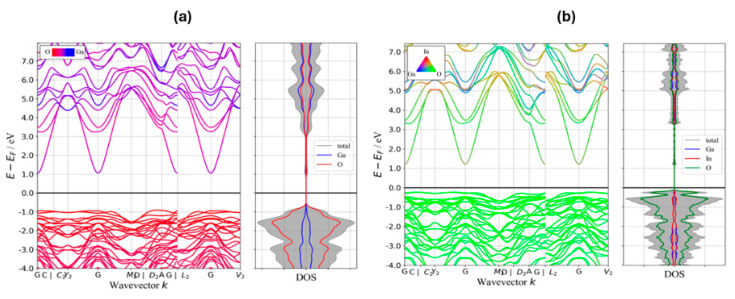
Band structure and density of states (DOS) for: (**a**) Ga_2_O_3_, (**b**) IGO.

Figure 7 depicts the band structure and density of states (DOS) for Ga_2_O_3_ and IGO with 50% substitution of In for Ga to illustrate the effect of adding In on the band gap and the features of the valence band (VB) and conduction band (CB). Band structures were obtained using GGA/PBE, and effective masses were extracted from parabolic fits to the band extrema. The band structure plot shows that the VB in both Ga_2_O_3_ and IGO is mostly comprising O states, while the CB of Ga_2_O_3_ and IGO is mostly formed from Ga/O and In/O states, respectively. As shown in Figure 7, adding In significantly increases the VBM and lowers the band gap. Because of the anisotropy of the conduction and valence bands, we report the directional effective masses along the principal high-symmetry paths (Γ→C, Γ→Y_2_, Γ→M_2_, Γ→A, Γ→L_2_, Γ→V_2_ for electrons) rather than a single scalar average. For Ga_2_O_3_, the electron effective masses (in units of m_0_) are: Γ→C 0.236, Γ→Y_2_ 0.262, Γ→M_2_ 0.262, Γ→A 0.219, Γ→L_2_ 0.262, and Γ→V_2_ 0.259. For the (Ga_0_._5_In_0_._5_)_2_O_3_ alloy, the corresponding electron masses are Γ→C 0.211, Γ→Y_2_ 0.236, Γ→M_2_ 0.239, Γ→A 0.204, Γ→L_2_ 0.239, and Γ→V_2_ 0.233. These calculations indicate that the incorporation of In into Ga_2_O_3_ significantly reduces the bandgap and decreases the electron effective mass. Overall, the DFT results support the experimental finding that In alloying reduces the band gap and electron effective mass, thereby facilitating higher electron mobility in IGO alloys.

## 4. Conclusions

The structural, optical, and electrical properties of MOCVD-grown Indium gallium oxide (IGO) films were thoroughly studied by varying the indium composition. The experimental results demonstrate that the bandgap of the films can be effectively tuned with indium incorporation, with a relatively smooth variation despite the corresponding changes in crystal structure. The films exhibit two distinct crystal phases: monoclinic and bixbyite, depending on the percentage of indium in the film. Most of the as-grown films exhibit high resistivity, whereas pure In_2_O_3_ displays high p-type conductivity with a large free carrier density. The influence of hydrogen on the electrical properties of the IGO films was also examined, revealing that hydrogen significantly enhances the free carrier density by acting as a shallow donor. Finally, DFT calculations confirm that the primary effect of In alloying on the band structure of Ga_2_O_3_ is to reduce both the band gap and the effective mass of electrons.

## Figures and Tables

**Table 1:** Interplanar spacing calculated with Bragg’s equation for -201 and 222 planes with different Indium and Gallium percentages of IGO.

Sample	-201 Peak Position (2θ)	Interplanar Spacing (d_-201_) (d = λ/2sinθ)	Sample	222 Peak Position (2θ)	Interplanar Spacing (d_222_) (d = λ/2sinθ)
Pure Ga_2_O_3_	19.42^o^	4.57 Å	Pure In_2_O_3_	31.04^o^	2.88 Å
IGO (In 20%)	19.02^o^	4.66 Å	IGO (In 80%)	31.30^o^	2.85 Å
IGO (In 40%)	18.89^o^	4.70 Å	IGO (In 60%)	31.50^o^	2.83 Å

**Table 2:** Electrical parameters of the films grown on sapphire substrate (N/M = Not measurable).

Sample Name	Resistivity (Ohm·cm)	Mobility (cm^2^/Vs)	Density (cm^−3^)	Hall Coeff. (cm^3^/Coul)	Sheet Number (cm^−2^)	Sheet Res. (Ohm/cm^2^)
Pure Ga_2_O_3_	1.01 × 10^4^	<1	3.02 × 10^10^	2.00 × 10^8^	3.02 × 10^6^	1.02 × 10^8^
IGO (In 20%)	4.00 × 10^4^	<1	2.92 × 10^9^	2.14 × 10^9^	2.91 × 10^5^	4.09 × 10^8^
IGO (In 40%)	9.00 × 10^5^	<1	6.07 × 10^8^	1.03 × 10^10^	6.06 × 10^4^	9.00 × 10^9^
IGO (In 60%)	N/M	N/M	N/M	N/M	N/M	N/M
IGO (In 80%)	N/M	N/M	N/M	N/M	N/M	N/M
Pure In_2_O_3_	0.65	15.69	6.15 × 10^17^	10.15	6.15 × 10^13^	6.47 × 10^3^

**Table 3:** Electrical parameters of the samples after hydrogen diffusion at 400 °C.

Sample Name	Resistivity (Ohm·cm)	Mobility (cm^2^/Vs)	Density (cm^−3^)	Hall Coeff. (cm^3^/Coul)	Sheet Number (cm^−2^)	Sheet Res. (Ohm/cm^2^)	Type of Carriers
IGO (In 20%)	0.59	2.37	4.48 × 10^18^	1.39	4.48 × 10^14^	5885.142	electrons
IGO (In 80%)	8.87 × 10^−2^	15.2414	4.62 × 10^18^	1.35239	4.62 × 10^14^	887.3159	electrons

## Data Availability

The original contributions presented in this study are included in the article. Further inquiries can be directed to the corresponding author.
